# Unveiling local molecular desorption dynamics using higher-order optical resonances

**DOI:** 10.1007/s12200-025-00159-1

**Published:** 2025-07-28

**Authors:** Mingquan Deng, Xiujie Dou, Xiaoyu Wang, Yin Yin, Xun Guan, Libo Ma, Xing Ma, Jiawei Wang

**Affiliations:** 1 School of Integrated Circuits, Harbin Institute of Technology (Shenzhen), Shenzhen, 518055 China; 2 School of Physics and Optoelectronic Engineering, Hainan University, Haikou, 570228 China; 3 School of Materials Science and Engineering, Jiangsu University, Zhenjiang, 212013 China; 4 Tsinghua Shenzhen International Graduate School, Tsinghua University, Shenzhen, 518055 China; 5 Leibniz IFW Dresden, Dresden, 01069 Germany

**Keywords:** Microtube cavity, Whispering-gallery-mode (WGM), Molecular desorption, High-order axial mode, Optical sensing

## Abstract

Understanding the sorption dynamics between water molecules and various solid surfaces is of great interest in diverse fundamental and industrial research. For studying such dynamics in a microsystem, existing investigations mainly focus on sorption behaviors mediated by external temperature variations. Here, we demonstrate a route to in situ sensitive detection of laser irradiation-induced localized water molecule desorption at a sub-monolayer level on an oxide surface. Harnessing a tailored set of optical whispering-gallery-mode (WGM) resonances in a nanomembrane-based microtube cavity, the desorption can be tracked by resonance mode shift in real-time, and further explained using a combination of pseudo-first-order and pseudo-second-order models. Additionally, upon adjusted laser excitation locations, the axial-mode-dependent responses enable the retrieval of corresponding profiles of desorption-induced perturbation at equilibrium. This study provides new insights into molecular desorption kinetics and introduces a spatially resolved sensing technique with applications in surface science, molecular sensing, and the study of desorption dynamics at the nanoscale.

**Graphical Abstract:**

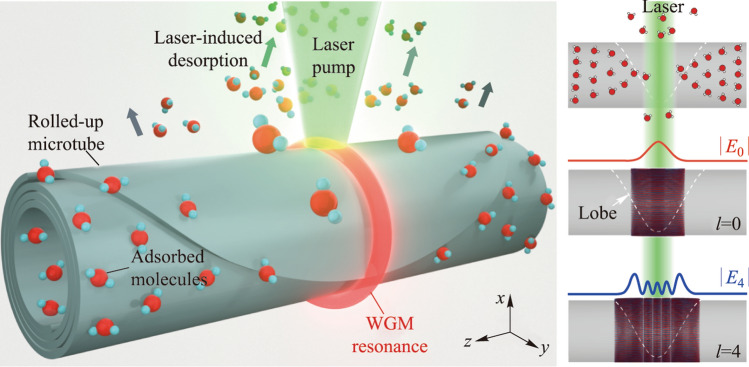

## Introduction

Understanding the interactions between water molecules and solid surfaces, such as sorption, diffusion, and permeation, essentially forms the foundation of various fundamental and applied research areas, such as surface and interface physics [1–4], biophysics [5], and catalysis [6, 7]. Nowadays, various label-free optical sensing technologies, leveraging interference or resonance phenomena, have been extensively investigated for high-sensitivity detection of target molecules in both aqueous [8–11] and air environments [12, 13]. A large variety of optical approaches have proven effective for measuring bulk water content (e.g., refractive index sensing) [14, 15] and humidity [16–18]. In contrast to bulk sensing, when significantly fewer water molecules interact with sensors, one should consider molecular properties and morphologies rather than macroscopic refractivity. Typically, the characterization of water molecules on solid surfaces at the sub-monolayer level was conducted using scanning tunneling microscopy (STM) [19], and low-energy electron diffraction (LEED) [20], whereas the demonstration using optical sensors remains elusive.

Among the developed label-free optical sensing platforms, whispering-gallery-mode (WGM) microcavities have garnered significant interest due to their ability of tight optical confinement strong light-analyte interaction, offering detection limits down to the single-molecule level (e.g., DNAs [21, 22], viruses [23], and proteins [10, 24]). The versatility of WGM-based optical sensors has been demonstrated across a range of microcavity geometries, including microspheres [12, 15, 25, 26], microdisks [27, 28], microrings [29–31], microtoroids [18, 32–34], and microtubes [13, 14, 35–38]. Among these, microtubular-shaped WGM, particularly those fabricated via nanomembrane origami techniques, stand out due to their hollow-core architecture with sub-wavelength-thick cavity walls, offering superior surface sensitivity to molecular adsorption and desorption [39]. Besides, the flexibility of fabricating nanomembrane-based WGM sensors from diverse materials (e.g., oxides and semiconductors) makes them ideal for probing fine interactions between various non-conductive surfaces and small molecules such as water [37, 38, 40], ethanol [15, 38], and volatile organic compounds (VOCs) [12]. In our prior work [38, 41], the dynamics of water molecular adsorption and release on the oxide surface, as well as the corresponding morphologies of water nanostructures, have been revealed using WGM resonances in nanomembrane-based WGM sensors. However, these investigations primarily relied on external thermal control (e.g., leveraging a liquid helium cryostat [36]), leaving the influence of light irradiation (e.g., via a tightly-focused laser beam) on these interactions largely underexplored.

In this work, the laser-triggered interaction between water molecules and oxide surfaces is unveiled using sculptured three-dimensional (3D) resonances in a rolled-up microtube formed with a tailored nanomembrane design. By continuously monitoring the mode blueshift in the WGM resonant photoluminescence (PL) spectrum, we achieve in situ tracking of the laser-triggered desorption of water molecules at the hydrophobic oxide surface with sub-monolayer level. Compared with conventional WGMs mostly confined into a plane, here tracking of 3D higher-order axial resonance modes enables spatially resolved molecular sensing in real-time, which reflects the profile of perturbation from local laser irradiation. Our proposed approach provides new insights into molecular dynamics at the water/solid interface, offering a powerful tool for studying water-related phenomena and the impact of local light irradiation on molecular processes.

## Results

### Working principle

**Fig. 1:**
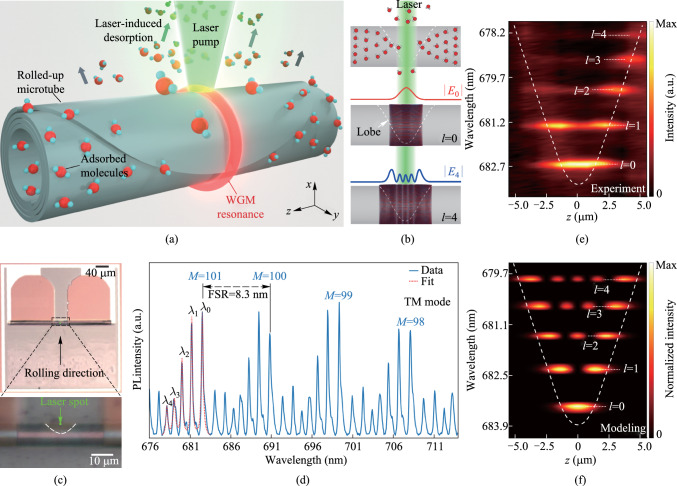
**a** Schematic illustration of probing laser irradiation-induced water molecule desorption via monitoring the mode shift. **b** Schematics showing the localized laser heating and triggered molecular desorption along the axial axis (top), and the corresponding distribution of the fundamental axial mode (middle, *l* = 0) and the fourth-order axial mode (bottom, *l* = 4). The curves denote the electric field intensity distributions. **c** Bright-field optical microscope images of the microtube cavity under 10 × (top) and 50 × (bottom) magnification, with the white dashed line outlining the lobe structure. **d** Measured resonant spectrum (in the range of *M* = 98 − 101) showing higher-order axial modes. The resonant wavelengths of discernable axial modes (*M* = 101) are denoted as *λ*_0_ – *λ*_4_, respectively. **e** Experimentally measured spatial distribution of axial modes (*l* = 0 − 4). **f** Numerically modeled spatial distribution of axial modes (*l* = 0 − 4). The white dashed lines indicate the optical quasi-potential well

Figure 1a and b illustrates the detection process of water molecule desorption, utilizing WGM resonant light emission from a rolled-up nanomembrane microtube cavity. The microtube is fabricated via a well-established nano-origami rolling technique [42], which allows precise control over key structural parameters, including tube size, wall thickness, and winding number. The primary advantage of the technique lies in its ability to engineer resonance profiles along the axial dimension via tailoring the 2D planar nanomembrane geometry into an extruded “lobe” design [43–46]. The presence of the lobe effectively forms an optical quasi-potential well, splitting the original WGM resonance into multiple higher-order axial modes [47–50]. In contrast to the widely-studied microbottle resonators supporting 3D axial resonances [47], the microtube resonators empowered by nanomembrane origami technique feature ultra-thin cavity wall thickness down to several tens of nanometers. Consequently, strong evanescent fields of the set of distinct axial modes are supported at the cavity surface, facilitating the detection of water molecule sorption dynamics [38, 40]. By incorporating defects or gain materials [37] into the dielectric nanomembrane, WGM emission can be excited via optical pumping using a focused laser beam. Given the localized heating due to focused pumping (with a spot size of ~ 1 μm^2^), higher-order axial modes exhibit distinct responses due to the varying spatial overlap between the resonant fields and the perturbed area due to laser irradiation (see Fig. 1b). By analyzing the responses of the mode set measured simultaneously and applying perturbation theory [51], the associated desorption profile can be determined.

In experiments, silicon nitride (SiN_*x*_) microtubes are fabricated using our latest dry-release-based nanomembrane origami technique (see Fig. 1c). The center portion of the 2D nanomembrane was tailored into a parabolic-like shape upon lithography and etching (see Methods, Sect. 5). Due to the amorphous nature of the deposited SiN_*x*_ nanomembrane film [42, 52, 53], defects-induced broadband PL between 600 and 800 nm gets excited and coupled into WGMs while optically pumped with a 532 nm laser. The measured resonant spectrum in Fig. 1d reveals distinct axial mode families for each mode group defined with different azimuthal mode number *M*, ranging from the fundamental mode (with axial mode number *l* = 0) to the fourth higher-order mode (*l* = 4). Given the subwavelength thickness of the microtube wall (~ 250 nm), only transverse-magnetic (TM) polarized WGM resonances (with the electric field oriented parallel to the microtube axis) are well-supported [15]. To further investigate the spatial distribution of these higher-order axial modes, spatially resolved measurements were performed (see Methods, Sect. 5). In Fig. 1d, the difference in mode antinode numbers clearly corresponds to the axial mode order. Based on numerically solving the quasi-Schrödinger equation for the lobe-induced optical quasi-potential well, theoretical calculations in Fig. 1f show excellent agreement with the measured mode field distributions (see Fig. 1e).

### In situ characterizations of water desorption

**Fig. 2:**
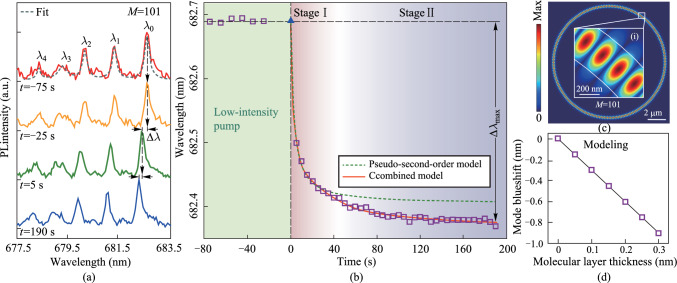
**a** Temporal evolution of resonance spectra under varying laser excitation intensities at the center of the lobe (*z* = 0 μm). Gray line: multi-peak Lorentzian fit to extract the resonant wavelengths for each axial mode. **b** Summarized temporal variation of the resonant wavelength for the fundamental mode. Lines: fits based on the pseudo-second-order model (green, *k*_2_ = − 1.30 s^−1^) and the combined model (orange, *k*_2_ = − 1.30 s^−1^, *k*_1_ = − 4.55 s^−1^). **c** Simulated mode field intensity distribution of the cross-section of a microtube cavity. Inset: Zoomed-in view. **d** Mode shift as a function of the equivalent released molecular layer thickness derived using perturbation theory

In the sensing experiment, the group of axial modes around 677.5 − 683.5 nm with the same azimuthal mode number *M* = 101 is tracked (see Fig. 2a), enabling the resonant wavelengths to serve as in situ probes for molecular desorption-induced perturbations. Initially, the laser intensity is set to a low value using a neutral density (ND) filter (optical density = 1) (see *t* = − 80 s to 0 s in Fig. 2b), and the resonant wavelength of the fundamental mode remains highly stable in thermal equilibrium with negligible heating effects under low laser excitation power. In Fig. 2a, this behavior is consistent across all axial modes. At *t* = 0 s, the laser power was increased tenfold. One can clearly discern a continuous blueshift of the resonant modes, which is attributed to the laser irradiation-induced desorption of water molecules from the microtube surface. Given the nature of weak perturbation, the magnitude of the mode shift $\Delta \lambda$ can be expressed using perturbation theory [38, 41, 51] based on the following formula:

1 $$\frac{\Delta \lambda }{\lambda } \approx \frac{\Delta \omega }{\omega } = - \frac{{\left\langle {E\left( {\vec{r}} \right)} \right.\left| {\Delta \varepsilon \left( {\vec{r}} \right)} \right|\left. {E\left( {\vec{r}} \right)} \right\rangle }}{{2\left\langle {E\left( {\vec{r}} \right)} \right.\left| {\varepsilon \left( {\vec{r}} \right)} \right|\left. {E\left( {\vec{r}} \right)} \right\rangle }},$$

where $\lambda$, $\omega$, $E\left(\overrightarrow{r}\right)$, and $\varepsilon \left(\overrightarrow{r}\right)$ are the resonance wavelength, resonance angular frequency, the electric field distribution in the resonator, and the permittivity, respectively. Here $\Delta \varepsilon \left(\overrightarrow{r}\right)$ denotes the permittivity variation induced by the change of absorbates.

According to the calculation of the perturbation theory, $\Delta \lambda /\lambda$ is linearly proportional to the amount of desorbed water molecules proportion $\Delta q/q$ (or effectively the molecular layer thickness assuming a uniform coverage). In Fig. 2b, for the fundamental mode, the blueshift saturates at ~ − 0.31 nm by *t* = 190 s under continuous laser-induced heating. Figure 2c presents the simulated mode field distribution at *M* = 101 through finite element method. Numerical calculations in Fig. 2d indicate that this shift corresponds to the desorption of a ~ 0.1-nm-thick water layer (corresponding to 33% of a monolayer). The limited strength of desorption is attributed to the hydrophobic nature of the Al_2_O_3_ surface. Considering the spectral resolution of ~ 20 pm, the estimated noise-equivalent detection limit can be down to ~ 2% change of a water monolayer.

To further analyze the desorption dynamics, we apply the statistical rate theory (SRT) as a widely adopted approach to describe the kinetic behavior of adsorbates (e.g., water molecules) on the solid surface in a gas/solid or solution/solid system [54–57]. The desorption rate is expressed in its general form as [56]:

2 $$\frac{{{\text{d}}q\left( t \right)}}{{{\text{d}}t}} = K_{ls} c_{e} \left( {1 - \frac{{q_{e} }}{{q_{m} }}} \right)\left[ {K_{L} c\left( {\frac{{q_{m} - q\left( t \right)}}{q\left( t \right)}} \right)^{{{1 \mathord{\left/ {\vphantom {1 \nu }} \right. \kern-0pt} \nu }}} - \frac{1}{{K_{L} c}}\left( {\frac{q\left( t \right)}{{q_{m} - q\left( t \right)}}} \right)^{{{1 \mathord{\left/ {\vphantom {1 \nu }} \right. \kern-0pt} \nu }}} } \right],$$

where *q*(*t*) is the amount of adsorbates on the solid surface at time *t*, *q*_*e*_ is the amount at final equilibrium, *q*_*m*_ is the monolayer capacity, *K*_*ls*_ is the reaction rate constant, and *K*_*L*_ is the system equilibrium constant. Here *c* denotes the concentration, *c*_*e*_ is the concentration at the equilibrium, and *ν* is the heterogeneity parameter ranging from 0 to 1 (with *ν* = 1 corresponding to the ideally homogeneous surface).

Given the complexity, the SRT model is often simplified into the pseudo-first-order and pseudo-second-order models [58, 59]. In Fig. 2b, for *t* = 0 − 30 s, we define the period exhibiting a rapid and intense mode blueshift as “Stage I”. The fast response is attributed to the primary desorption, which can be well fitted via a single pseudo-second-order kinetic model:

3 $$\frac{\Delta q\left( t \right)}{{q_{0} }} = \frac{{q\left( t \right) - q_{0} }}{{q_{0} }} = \frac{{q_{e} - q_{0} }}{{q_{0} }} - \frac{1}{{k_{2} t + {{q_{0} } \mathord{\left/ {\vphantom {{q_{0} } {\left( {q_{e} - q_{0} } \right)}}} \right. \kern-0pt} {\left( {q_{e} - q_{0} } \right)}}}} = \frac{{\Delta q_{\max } }}{{q_{0} }} - \frac{1}{{k_{2} t + {{q_{0} } \mathord{\left/ {\vphantom {{q_{0} } {\Delta q_{\max } }}} \right. \kern-0pt} {\Delta q_{\max } }}}},$$

where $\Delta q(t)$ is the amount of desorbed molecules at time *t*, *q*_0_ is the initial amount of adsorbates, ${\Delta q}_{\text{max}}$ is the accumulated amount at final equilibrium, and *k*_2_ is the desorption time scale factor, which depends on the initial concentration and the system temperature. The fit in Fig. 2b yields *k*_2_ = − 1.30 s^−1^, ${\Delta q}_{\text{max}}/{q}_{0}$ of − 0.29. Notably, for *t* > 30 s, the tracked mode shift gradually deviates from the prediction using the pseudo-second-order model, suggesting the potential contribution of an additional mechanism. In previous studies, the pseudo-first-order kinetic model has been employed for the prediction of molecular dynamics near equilibrium [56]. Here for *t* = 30 − 190 s, we define the period exhibiting a smooth and moderate mode blueshift as “Stage II”. Due to its distinct behavior compared to Stage I, we propose a combined model incorporating contributions from both pseudo-first-order and pseudo-second-order dynamics:

4 $$\frac{\Delta q\left(t\right)}{{q}_{0}}={w}_{1}\left(\frac{\Delta {q}_\text{max}}{{q}_{0}}-\frac{\Delta {q}_\text{max}}{{q}_{0}}e^{-{k}_{1}t}\right)+{w}_{2}\left(\frac{\Delta {q}_\text{max}}{{q}_{0}}-\frac{1}{{k}_{2}t+{q}_{0}/{\Delta {q}_\text{max}}}\right),$$

where *k*_1_ is the desorption time scale factor of the pseudo-first-order dynamics, and *w*_1_ and *w*_2_ are the weighting factors of two mechanisms. The fit for Stage II in Fig. 2b suggests a dominant contribution from the pseudo-second-order dynamics with *w*_2_ = 2.46, with a minor contribution from the pseudo-first-order dynamics (*w*_1_ = 0.73).

### Laser irradiation-induced desorption resolved by axial resonances

**Fig. 3:**
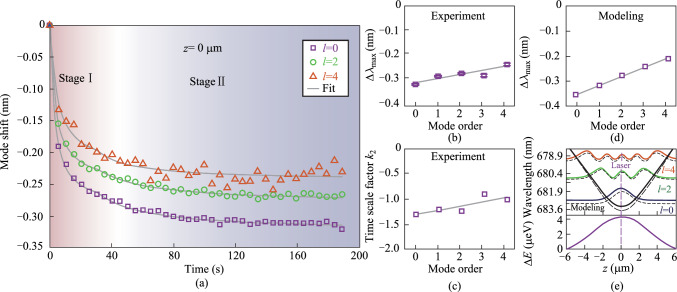
**a** Summarized temporal variation of the resonance wavelength shifts for three axial modes (*l* = 0, 2, and 4) under high laser intensity focused at the center of the lobe (*z* = 0 μm). **b** Summarized Δ*λ*_max_ as a function of the axial mode order. **c** Summarized time scale factor *k*_2_ as a function of the axial mode order. **d** Numerically calculated Δ*λ*_max_ using perturbation theory. The gray lines in **b** and **d** serve as visual guides. **e** Top: Numerically calculated spatial distribution of axial modes (*l* = 0, 2, and 4) at the original state (dashed lines) and final equilibrium (solid lines) due to laser radiation. The black lines denote the boundary of the optical quasi-potential wells at the original state (dashed line) and final equilibrium (solid line). Bottom: corresponding laser irradiation-induced perturbation on the potential well. The purple dotted line denotes the location of laser irradiation

Given the laser irradiation is predominantly localized to the micrometer scale, the mode-dependent response is studied by tracking the mode shift of all discernable axial modes with the same *M* (101 as an example). Initially, the laser spot is aligned at the lobe center (*z* = 0 μm). As illustrated in Fig. 3a, the tracked mode shift for three axial modes with *l* = 0, 2 and 4 reveal distinct desorption rates and saturation levels while reaching the equilibrium. As summarized in Fig. 3b, the lower-order modes, more concentrated around the center of the lobe (see Fig. 1e), experience a stronger perturbation, and consequently a larger mode blueshift. Meanwhile, the mode shift at the initial stage is analyzed by fitting using Eq. (3). The fundamental mode features the lowest value of *k*_2_ of − 1.30 s^−1^, indicating the fastest molecular desorption. In contrast, higher-order axial modes exhibit slightly prolonged desorption processes.

At the final equilibrium (*t* ~ 190 s), the mode-dependent responses can be interpreted as spatially-resolved in situ detection of molecular dynamics. By modeling the laser-induced desorption as a Gaussian distribution along the axial direction, we find that the desorbed water molecules induce a global modification of the optical quasi-potential well. Setting the lateral dimension of the irradiation-induced perturbation Δ*λ* to ~ − 0.31 nm, one can numerically calculate the perturbed axial modes, including resonant wavelengths and the spatial mode field distributions (see Fig. 3d). The trend of extracted Δ*λ*_max_ in the calculation is consistent with the experimental observations in Fig. 3b.

**Fig. 4:**
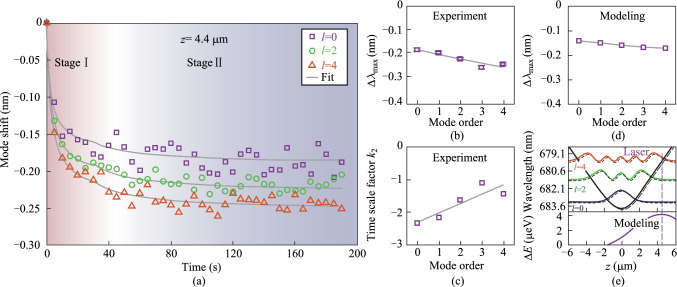
**a** Summarized temporal variation of the resonance wavelength shifts for three axial modes (*l* = 0, 2, and 4) under high laser intensity with the focus shifted away from the center of the lobe (*z* = 4.4 μm). **b** Summarized Δ*λ*_max_ as a function of the axial mode order. **c** Summarized time scale factor *k*_2_ as a function of the axial mode order. **d** Numerically calculated Δ*λ*_max_ using perturbation theory. The gray lines in **b** and **d** serve as visual guides. **e** Top: Numerically calculated spatial distribution of axial modes (*l* = 0, 2, and 4) at the original state (dashed lines) and final equilibrium (solid lines) due to laser radiation. The black lines denote the boundary of the optical quasi-potential wells at the original state (dashed line) and final equilibrium (solid line). Bottom: corresponding laser irradiation-induced perturbation on the potential well. The purple dotted line denotes the displaced position of laser irradiation

To further investigate the axial mode-dependent responses during molecular desorption, additional experiments were conducted with the laser spot displaced from the lobe center to *z* = 4.4 μm, while maintaining the same experimental configuration. As shown in Fig. 4a, all three discernable modes exhibit mode blueshift similar to those observed in Fig. 3a. Here the signal–noise ratio, particularly for the fundamental mode, becomes degraded, due to the weakened signals collected by the objective lens at the shifted axial position. The corresponding mode shift for the fundamental mode is less pronounced, decreasing significantly to − 0.19 nm compared to − 0.31 nm in Fig. 3a. In contrast, the higher-order axial mode *l* = 4 suggests the largest mode shift, reaching up to − 0.25 nm. This result highlights the strongest spatial overlap between the laser focus and the mode field intensity maximum for *l* = 4, which occurs at approximately 4.4 μm, as predicted by the calculated mode field distributions in Fig. 1e.

Consistent with the finding of the time scale factor *k*_2_ in Fig. 3, here, the fundamental mode again suggests the fastest response with *k*_2_ of − 2.34 s^−1^. For higher-order modes, the enhanced disparity *k*_2_ between each mode may arise from the difference in local irradiation strength, which gradually decays from the right side (*z* > 0) across the center to the left side (*z* < 0), while the spatial inhomogeneity results in distinct rates of reaching local dynamic equilibrium. In numerical calculations, by adopting a shifted location to *z* = 4.4 μm for setting the irradiation-induced perturbation, the quasi-potential well gets deformed as a whole. Furthermore, the resulting Δ*λ*_max_ for all axial modes shows a nice agreement with the observation in Fig. 4b.

## Conclusion

In summary, we have experimentally demonstrated the detection of surface molecular dynamics using tailored 3D WGM resonances in a nanomembrane-based tubular cavity. Unlike previous studies that primarily focused on molecular adsorption/desorption processes over large variations of the environmental temperature, here our in situ characterization of mode shifts from WGM emission reveals the dynamics of laser irradiation-triggered, local desorption of water molecules from oxide surfaces, achieving a resolution at sub-monolayer level. The axial-mode-dependent responses, together with theoretical analysis based on perturbation theory, elucidate the profile of localized perturbation upon the varying irradiation location and corresponding perturbed optical quasi-potential well. This work provides new insights into the thermal desorption kinetics of water molecules and their spatially resolved interactions on hydrophobic oxide thin films. Besides, it is envisaged that the technology can be further applied to various types of irradiation-mediated molecular dynamics such as photodegradation, biological transformation, and protein–protein interactions, offering implications for both fundamental studies and practical applications in surface science and molecular sensing.

## Methods

### Device fabrication

The devices were fabricated using a dry-etching-based nanomembrane origami technique, as described in our previous work [42]. The composite nanomembrane, consisting of an Al_2_O_3_ stop layer, a Si sacrificial layer, an Al_2_O_3_ protection layer, a SiN_*x*_ strained layer, and another Al_2_O_3_ protection layer was prepared on a silicon wafer via chemical vapor deposition (for SiN_*x*_ and Si) and atomic layer deposition (for Al_2_O_3_). A photoresist layer (AZ-5214E, Microchemicals GmbH, Germany) was spin-coated onto the nanomembrane and patterned using a maskless lithography (MLA 100, Heidelberg Instruments Mikrotechnik GmbH). Reactive ion etching (RIE, Plasma Laboratory 100; Oxford Instruments PLC, Abingdon, UK) was used to etch the patterned trenches into the sacrificial layer. The entire sample was then passivated with an Al_2_O_3_ thin film. After opening a release window to define the starting rolling edge, the strained nanomembrane self-assembled into microtubular structures through strain relaxation in a xenon difluoride etching system (Xactix e2 Orbotech Ltd., Yavne, Israel), followed by an additional Al_2_O_3_ passivation layer. By adjusting the stress condition of SiN_*x*_ layer, the diameter of the microtube could be well controlled within the range of ~ 10 − 20 μm. A U-shaped design was employed to ensure a gap between the center of the microtube and the substrate, effectively reducing the loss of microcavity WGM leakage to the substrate, achieving a quality factor (Q) of ~ 2000.

### Optical characterizations

Resonant spectra of the microtube cavities were collected using a confocal photoluminescence setup (LabRAM HR Evolution, HORIBA Scientific) following the same procedure in our previous study [15]. The laser spot displacement was adjusted using a motorized stage, which translated the microtube cavity under focused irradiation. A 532 nm continuous-wave laser (Cobolt Samba) was employed to generate a focused spot with a diameter of ~ 1 μm^2^ through a long-distance working objective (Olympus LMPLFLN 50 × ; numerical aperture = 0.5). The laser intensity was adjusted using a neutral density filter wheel. PL signals were collected using the same objective lens, and measured using a spectrometer equipped with a 600 blz/mm grating and an electrically cooled charge-coupled device (CCD) camera. The spatial distributions of axial modes were mapped by translating the microtube cavities from − 6 to 6 µm with a step of 0.1 μm along the *z*-axis through a motorized stage.

### Sensing tests

The chip containing integrated microtube cavities was mounted in a custom-designed chamber with a volume of ~ 0.7 cm^3^. The chamber was constructed with a substrate, a spacer, and a 0.17-mm-thick cover glass and sealed by ultraviolet-curing optical adhesives (NOA 68). A saturated K_2_SO_4_ solution was prepared and slowly delivered to the chamber using a syringe, offering a constant relative humidity of ~ 60% RH at room temperature (25 °C). The temporal resolution of the current spectral acquisition system is ~ 1 ms, enabling the tracking of fast transient processes. During the studies of laser-induced molecular desorption, the resonant spectra were repeatedly measured with a time resolution of 5 s, ensuring a high signal-to-noise ratio through sufficient integration time.

## Data Availability

The data that support the findings of this study are available from the corresponding authors, upon reasonable request.
